# Dynamics of Calcium during *In vitro* Microspore Embryogenesis and *In vivo* Microspore Development in *Brassica napus* and *Solanum melongena*

**DOI:** 10.3389/fpls.2017.01177

**Published:** 2017-07-07

**Authors:** Alba Rivas-Sendra, Antonio Calabuig-Serna, Jose M. Seguí-Simarro

**Affiliations:** Cell Biology Group, Institute for Conservation and Breeding of Valencian Agrodiversity (COMAV), Universitat Politècnica de ValènciaValencia, Spain

**Keywords:** androgenesis, eggplant, FluoForte, *in vitro* culture, microgametogenesis, microspore culture, microsporogenesis, rapeseed

## Abstract

Calcium is widely known to have a role as a signaling molecule in many different processes, including stress response and activation of the embryogenic program. However, there are no direct clues about calcium levels during microspore embryogenesis, an experimental process that combines a developmental switch toward embryogenesis and the simultaneous application of different stressing factors. In this work, we used FluoForte, a calcium-specific fluorescent vital dye, to track by confocal microscopy the changes in levels and subcellular distribution of calcium in living rapeseed (*B. napus)* and eggplant *(S. melongena)* microspores and pollen grains during *in vivo* development, as well as during the first stages of *in vitro*-induced microspore embryogenesis in rapeseed. During *in vivo* development, a clear peak of cytosolic Ca^2+^ was observed in rapeseed vacuolate microspores and young pollen grains, the stages more suitable for embryogenesis induction. However, the Ca^2+^ levels observed in eggplant were dramatically lower than in rapeseed. Just after *in vitro* induction, Ca^2+^ levels increased specifically in rapeseed embryogenic microspores at levels dramatically higher than during *in vivo* development. The increase was observed in the cytosol, but predominantly in vacuoles. Non-embryogenic forms such as callus-like and pollen-like structures presented remarkably different calcium patterns. After the heat shock-based inductive treatment, Ca^2+^ levels progressively decreased in all cases. Together, our results reveal unique calcium dynamics in *in vivo* rapeseed microspores, as well as in those reprogrammed to *in vitro* embryogenesis, establishing a link between changes in Ca^2+^ level and subcellular distribution, and microspore embryogenesis.

## Introduction

Microspores are the precursors of pollen grains, haploid gametophytes that will give rise to male gametes in plants. By applying specific stresses and conditions *in vitro*, microspores can be deviated from the original gametophytic pathway toward androgenesis, an experimentally induced embryogenic fate. These cells are then reprogrammed to become totipotent and start dividing sporophytically to produce a new haploid microspore-derived embryo (MDE). Either spontaneously or using genome duplication treatments, haploid MDEs may become doubled haploid (DH) and therefore 100% homozygous. This experimental process is highly interesting for both basic research and applied plant breeding. At the basic research level, the main interest of microspore embryogenesis resides on being an exceptional example of the fascinating developmental plasticity of plant cells. The change in cell fate undergone by microspores constitutes an excellent model to understand the cellular and molecular mechanisms underlying cell totipotency. Once the particular characteristics of this system are well-understood, it may serve as a model for the study of the very early stages of zygotic embryogenesis itself, overcoming the technical difficulties imposed by the maternal tissues surrounding the zygotic embryo.

In *Brassica napus* microspore cultures, embryogenic development starts from late uninucleated microspores and young bicellular pollen. A series of sporophytic divisions make the multicellular MDE grow inside the pollen exine, stretching and thinning it until the increasing size causes its rupture (Hause et al., 1994). MDEs that emerge from the exine follow the typical morphogenic pattern of zygotic embryos through globular, heart-shaped, torpedo, and cotyledonary stages, while non-induced microspores either arrest and die (Seguí-Simarro and Nuez, 2008), or continue a gametophytic-like development to become pollen-like structures which, after 5–6 days in culture, usually burst and therefore die (Soriano et al., 2013).

To be induced to embryogenesis, microspores of most species, including those of rapeseed, are subjected to a heat stress treatment. The first perception of heat stress occurs at the level of the plasma membrane via changes in its fluidity (Vigh et al., 1985; Horvath et al., 1998), which, together with the activation of stress-specific Ca^2+^-permeable channels, causes a transient increase in cytosolic Ca^2+^ levels (Liu et al., 2005). Elevation of cytosolic calcium levels is thought to be a primitive and universal response to stress (White and Broadley, 2003). As a response to specific Ca^2+^ perturbations, cells activate specific combinations of Ca^2+^ sensors (Ca^2+^-binding proteins). Binding to Ca^2+^ change their properties, which in turn modify the way they interact with target proteins, thereby altering many different aspects of cell physiology which, altogether, may result in stress tolerance and/or a developmental switch (White and Broadley, 2003). However, the roles and locations of calcium go far beyond being an intracellular messenger in the cytoplasm. Plant cells store calcium in different compartments, including the endoplasmic reticulum, nucleus, cell wall and vacuoles. At first, it was thought that the major source of stored calcium was the cell wall, where it is tightly bound to pectins and plays a key role in cell wall physiology (Demarty et al., 1984). Recently, it was proposed that cell wall AGPs act as calcium capacitors to supply Ca^2+^ to the cytosol on demand (Lamport and Varnai, 2013). However, other studies pointed out that, regardless of the roles of other compartments as calcium stores, the major calcium store in plants appears to be the vacuole (Rudd and Franklin-Tong, 2001), where it acts as a counter-cation for different inorganic and organic anions (White and Broadley, 2003). From these compartments, calcium is mobilized to produce fluctuations in cytosolic Ca^2+^ levels that play key signaling and regulating roles in multiple physiological and developmental processes, including cell division and growth, stomatal closure, and response to several stresses, including pathogen attack and wounding (reviewed in White and Broadley, 2003). Using the potassium pyroantimonate technique, calcium distribution and levels have been assessed during anther development in different species. From these and other studies, it was deduced that calcium has a direct role in pollen development. Indeed, altered calcium distribution in anther walls and pollen in tobacco, wheat and rice leads to pollen abortion (Tian et al., 1998; Meng et al., 2000; Li et al., 2001), and gradients of Ca^2+^ are important to determine the polarity and location of pollen pores and growing pollen tubes (Tirlapur and Willemse, 1992). Later, during post-zygotic development, gradients of Ca^2+^ are important for the establishment of embryo polarity and seed germination (Hause et al., 1994).

Even in the context of the experimental induction of microspore embryogenesis, Ca^2+^ has been suggested to be either necessary or helpful for microspore induction as well as for MDE germination and conversion into plants. Experimenting with extracellular calcium concentrations and calcium signaling modulators, a relationship between calcium modulation and embryogenesis induction was proposed in rapeseed (Pauls et al., 2006), barley (Cho and Kasha, 1995) and bread wheat (Reynolds, 2000). Usually, stresses mobilize calcium stores, eliciting an increase in cytosolic free Ca^2+^ levels which, in turn, activates mitogen-activated protein kinase (MAPK) cascades, among other protein phosphatase/kinase cascades (Pearce and Humphrey, 2001). Interestingly, different components of MAPK cascades have been found differentially regulated during stress-mediated induction of microspore embryogenesis in rapeseed and tobacco (Coronado et al., 2002; Seguí-Simarro et al., 2005). Hause et al. (1994) studied the distribution of calmodulin and free cytosolic Ca^2+^ in globular and elongated MDEs, finding some degree of polarization in the latter. Calcium was also found helpful for conversion of rapeseed into plantlets when added to the culture medium in the form of CaCl_2_ (Tian et al., 2004). However, although its role in embryogenesis induction is suspected from indirect evidences and deductions, the knowledge about the precise role of calcium in this process is still very scarce and poorly understood. Perhaps due to the technical difficulties of detecting calcium in a complex *in vitro* system like this, nobody to our knowledge has been able to show how calcium distributes and accumulates during microspore embryogenesis. In this work we study the changes in levels and subcellular distribution of calcium during microspore/pollen *in vivo* development in rapeseed and eggplant (two species with high and recalcitrant response to microspore embryogenesis, respectively), and at different time points during the first stages of *in vitro*-induced microspore embryogenesis in rapeseed, establishing a link between calcium increases and microspore embryogenesis.

## Materials and methods

### Plant materials

Donor plants of rapeseed *(B. napus)* L. cv. Topas line DH4079 were grown in growing chambers of the COMAV Institute (Universitat Politècnica de València, Valencia, Spain). Plants were grown in 20 cm pots at 60% humidity and 16/8 photoperiod, 300 μE.m^−2^.s^−1^ light intensity, kept at 20°C until flowering and then transferred to 15°C. Donor plants of eggplant (*Solanum melongena*) of the high androgenic response line DH36 (Rivas-Sendra et al., 2017) were grown in 30 cm pots in greenhouses under controlled temperature (25°C) and natural light.

### *Brassica napus* microspore culture

Isolation, induction treatment and microspore culture were performed according to Custers (2003). Flower buds containing mostly late uninucleated microspores and early binucleated pollen were dissected from the plant, surface sterilized with 2% sodium hypochlorite for 10 min, and washed three times in sterile distilled water for a total of 15 min. Buds were crushed with a sterile syringe piston in NLN-13 medium. NLN-13 medium consist of NLN salts and vitamins as described by Nitsch and Nitsch (1967) supplemented with 13% sucrose and sterilized by filtration trhough a 0.22 μm filter. Microspores were isolated from the suspension by filtration through a 40 μm nylon mesh (Millipore) followed by three rounds of centrifugation at 100 g for 4 min each. Microspore density was calculated with a hemacytometer and adjusted to 4 × 10^4^ microspores/ml. The cellular suspension was plated, incubated in darkness for 3 days at 32.5°C to induce embryogenesis, and then at 25°C in darkness for embryogenesis progression.

### Callose, calcium, and FDA staining and detection

Rapeseed and eggplant microspores and pollen at different stages during microsporogenesis and microgametogenesis were stained with FluoForte (FF; Enzo Life Sciences, ENZ-52015) for calcium detection. Microspores and pollen were isolated from flower buds at different stages. To minimize the stress, the extraction process was quickly done and the solutions and plant material used were kept at 4°C. Excised buds were crushed with a syringe piston in phosphate-buffered saline (PBS), filtered through a 40 μm nylon mesh and centrifuged at 100 g for 4 min. The supernatant was discarded and the concentrated cell suspension was mixed with the same volume of 0.2 g/l FF in PBS (diluted from a 10 g/l stock solution of FF in DMSO), in order to achieve a 0.1 g/l working concentration. The suspension was incubated in darkness during 30 min. Then, cells were washed with 1 ml PBS and centrifuged in an Eppendorf centrifuge at 200 g for 2 min. The supernatant was discarded and pelleted cells were mounted in a microscope slide with Mowiol antifading mounting solution and immediately observed. Mowiol solution was prepared with 17% Mowiol 4–88 (Sigma-Aldrich) and 33% glycerol (v/v) in PBS. Rapeseed microspore cultures were prepared as described above, collected at different culture times and stained following the same protocol, with two modifications: samples and solutions were kept at room temperature and 0.2% aniline blue for callose detection was also added to the staining mix. Stained cells were mounted in a microscope slide with Mowiol antifading mounting solution and immediately observed. A minimum of 20 cells at each of the stages were carefully studied.

As a control of dye uptake, rapeseed microspores and pollen at different stages during microsporogenesis and microgametogenesis were stained with 0.001% fluorescein diacetate (FDA; Invitrogen, F-1303). Microspores and pollen were isolated from flower buds in PBS as described before, keeping solutions and plant material at 4°C to minimize the mortality. Stock solution of FDA was prepared in acetone at 0.2%, and 5 μl of the stock were added to 1 ml of cell suspension. Samples were incubated in darkness at 4°C during 10 min, then mounted in a microscope slide and immediately observed. In all cases, observations were carried out with a Zeiss LSM 780 confocal laser scanning microscope.

### Image analysis of fluorescence

Digital images were processed with Leica Application Suite Advanced Fluorescence (LAS AF) and FIJI software. Spectral imaging of mature pollen grains stained with FF was carried out in the confocal laser scanning microscope with laser excitation at 488 nm. A set of images was obtained, each image being acquired with a separate narrow bandwidth (8.9 nm), representing the complete spectral distribution of the fluorescence emission signals for every point of the image. The spectral analysis of defined areas and the visualization of images in coded colors depending on the emission spectrum was performed using the advanced linear unmixing function (LAS software), which separates mixed signals pixel by pixel using the entire emission spectrum of each defined fluorescent compound in the sample. Autofluorescence and FF signal were differentiated by comparison with the reference emission spectrum of FF provided by the manufacturer (www.enzolifesciences.com). Using Fiji software, different cell regions (vacuoles, cytosol+nucleus, and exine) were selected and their mean fluorescence intensity was measured.

## Results

### Optimization of FF staining conditions

FF has been successfully used in animal cells (Blaauw et al., 2012; Kim et al., 2013) but, to the best of our knowledge, has not yet been used to detect calcium in plant cells. In sperm or blood platelets, FF has been used at concentrations of 10–20 μM solved in the presence of Pluronic F-127 0.02–0.1% (Mendes-Silverio et al., 2012; Jansen et al., 2015). We performed a series of experiments in different conditions in order to optimize the staining protocol for our *in vivo* and *in vitro* microspores and pollen grains.

Pluronic F-127 is a non-ionic, surfactant polyol that facilitates the solubilization of water-insoluble dyes in physiological medium, and it has been used to help disperse acetoxymethyl (AM) esters of fluorescent ion indicators (Cohen et al., 1974). This is why it was suggested to maintain dye solubility and aid tissue penetration (Fricker et al., 2001). In our tests, we found that Pluronic F-127 (P2443, Sigma) 0.1% can be added to the working staining solution for improved solubility of the dye and increased stability of the solution over time, but we confirmed that, when used immediately after preparation, the addition of Pluronic F-127 to the staining mix made no difference in the staining efficiency. According to manufacturer's specifications, Pluronic F-127 might alter the membrane properties of the cell, so we decided to avoid its use.

In order to minimize the changes with respect to microspore culture conditions, we tried to use FF dissolved in NLN-13 culture medium (pH 5.8) instead of PBS. However, the staining of the cells was weak and irregular, and not all cells were homogeneously stained (data not shown), so we discarded its use. In order to optimize the working concentration of FF, preliminary tests were performed in root tips, where high levels of Ca^2+^ for xylem delivery are described in the extreme root tip and lateral root growing regions. We tested FF at 0.01, 0.05, 0.1, and 0.2 g/l. Fluorescence in root tips was only detectable when 0.1 or 0.2 g/l was used (Figure S1). Negative control without FF showed no signal. Next, we tested FF 0.05, 0.1, and 0.2 g/l in microspores. The intensity of green fluorescence, always keeping the same settings in the microscope and camera, was found to be proportional to FF concentration (Figure S2). Negative control without FF presented no signal (Figures S2A,A′). When FF was added, the intensity yielded by 0.05 g/l was too weak (Figures S2B,B′). The optimal fluorescence was found with the use of 0.1 g/l (Figures S2C,C′), while 0.2 g/l produced excessively saturated images (Figures S2D,D′). Thus, we established 0.1 g/l as the optimal working concentration for our cells.

**Figure 1:**
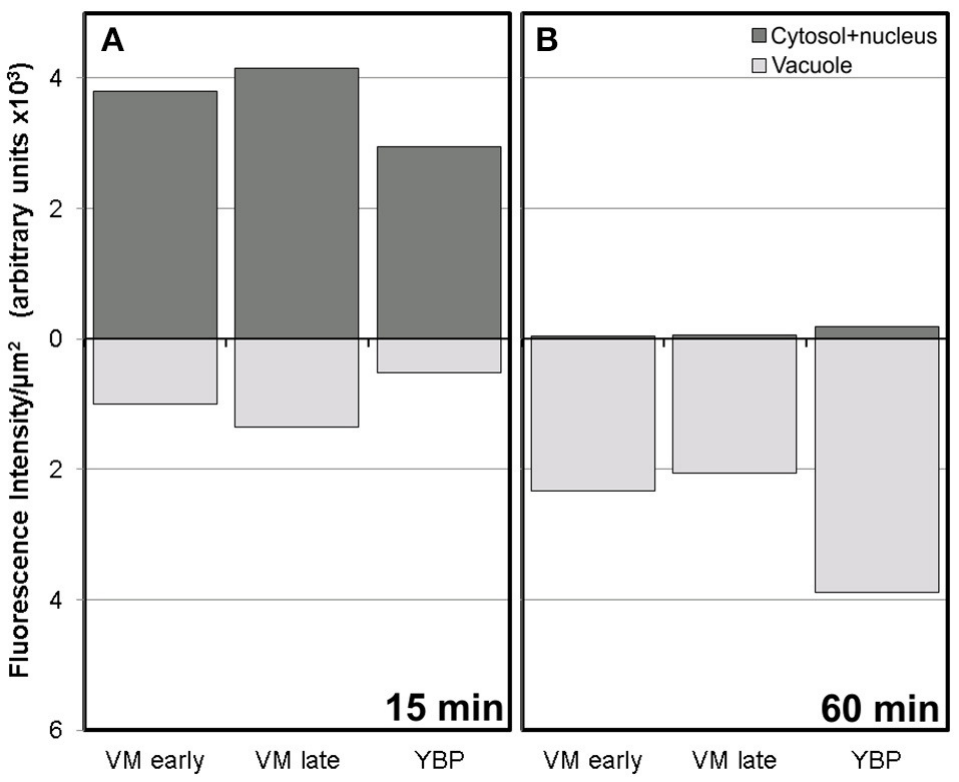
Fluorescence intensity of FF staining in the cytosol+nucleus region and in vacuoles of rapeseed vacuolate microspores and young bicellular pollen, 15 min **(A)** and 60 min **(B)** after mounting the samples.

**Figure 2:**
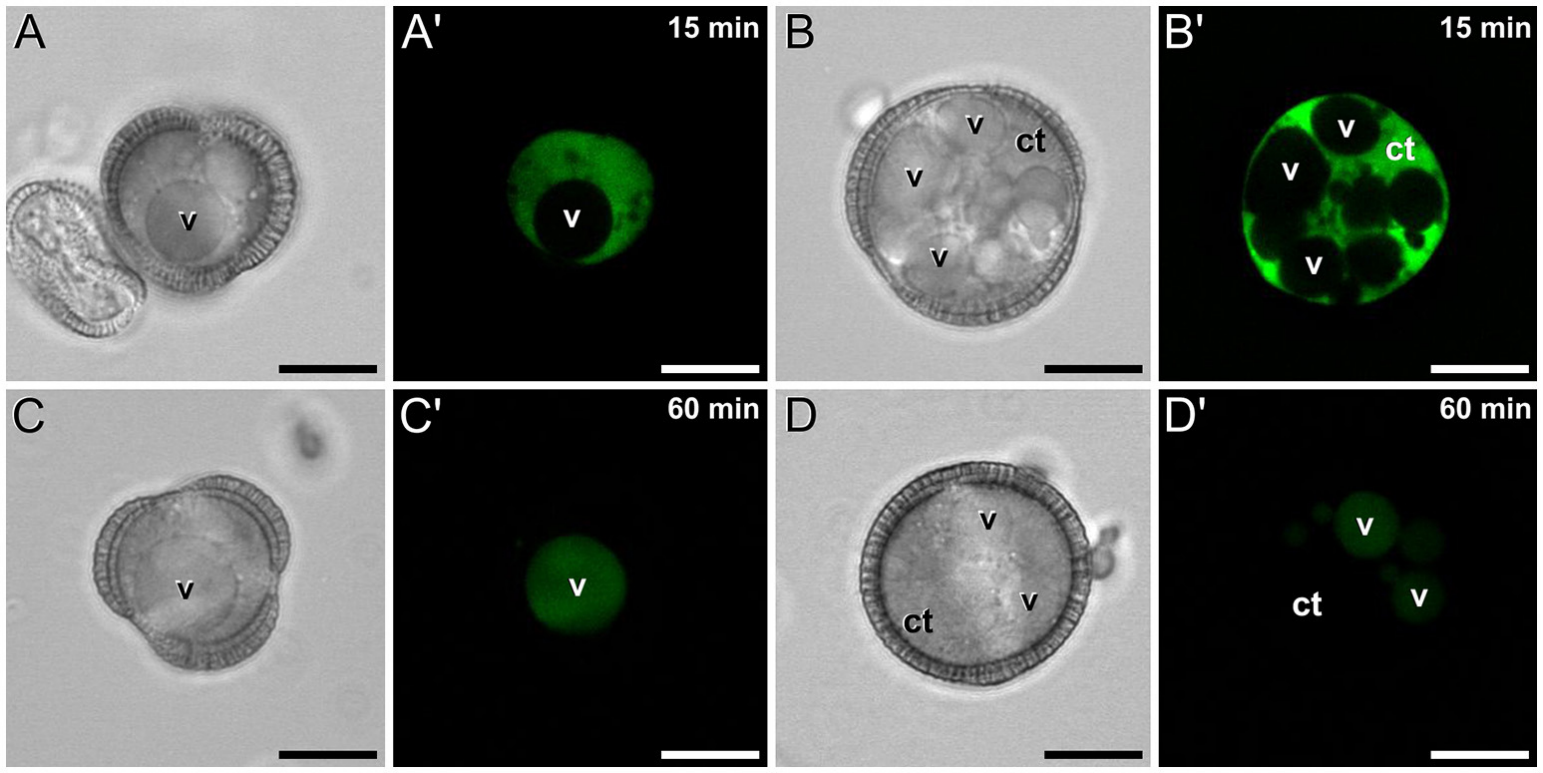
FF staining of rapeseed microspores and pollen after 15 and 60 min of observation. Phase contrast **(A–D)** and fluorescence **(A′–D′)** pairs of pictures are shown. Vacuolate microspore **(A,A′)** and early binucleated pollen **(B,B′)** after 15 min of observation. Vacuolate microspore **(C,C′)** and early binucleated pollen **(D,D′)** after 60 min of observation. Note the change of FF staining localization from nuclear-cytosolic to vacuolar in both cell types. Ct, cytosol; v, vacuole. Bars: 10 μm.

Conventional calcium-sensitive fluorescent dyes are known to undergo compartmentalization after prolonged incubation times (Fricker et al., 2001). Therefore, we studied the dynamics of FF staining in living, freshly isolated rapeseed microspores and pollen grains at different time points. Approximately 15 min after mounting samples in the slides (Figure 1A), both vacuolate microspores (Figures 2A,A′) and pollen grains (Figures 2B,B′) clearly showed an intense nuclear-cytosolic FF signal, having vacuoles a very low signal, barely eye-detectable. Prolonged observation of these cells revealed a progressive decrease of the nuclear-cytosolic signal and a parallel increase of the vacuolar signal. Thereby, 60 min after mounting (Figure 1B), the FF signal was almost excluded from the nucleus-cytosol and principally localized in the vacuoles. Such a transition from cytosolic to vacuolar signal was observed in both vacuolate microspores (Figures 2C,C′) and pollen grains (Figures 2D,D′). After 1 h, no further changes were observed. Quantitatively, the nucleus-cytosol/vacuole ratio averaged for the three stages shown in Figure 1 was 4.2 (four-fold more signal in the nucleus + cytosol than in vacuole) when observed within 15 min, and 0.03 (30-fold less signal in nucleus-cytosol than in vacuole) when observed after 60 min. Next, we checked eggplant microspores and pollen grains 15 and 60 min after mounting and observed exactly the same results (data not shown). Thus, we concluded that prolonged incubation times caused a progressive loss of nuclear-cytosolic signal together with a compartmentalization of the FF signal into vacuoles. However, observation of cells around 15 min after incubation and mounting consistently showed a clear nuclear-cytosolic signal in these cells. As a result of this, we assumed this time point as the optimal to reliably detect calcium with FF in our cells.

### Calcium distribution during microspore and pollen development in rapeseed

**Figure 3:**
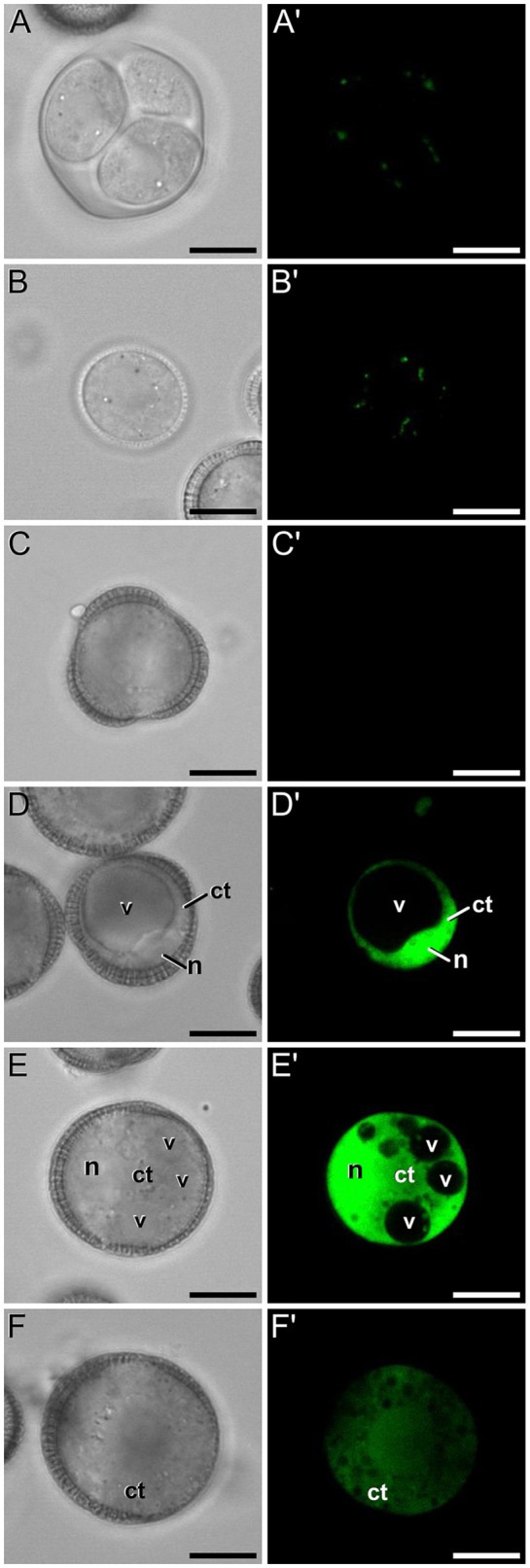
FF staining during *in vivo* microspore/pollen development in rapeseed. Phase contrast **(A–F)** and fluorescence **(A′–F′)** pairs of pictures of a tetrad **(A,A′)**, young microspore **(B,B′)**, mid microspore **(C,C′)**, vacuolate microspore **(D,D′)**, young bicellular pollen **(E,E′)**, and mid pollen grain **(F,F′)** are shown. ct, cytosol; n, nucleus; v, vacuole. Bars: 10 μm.

**Figure 4:**
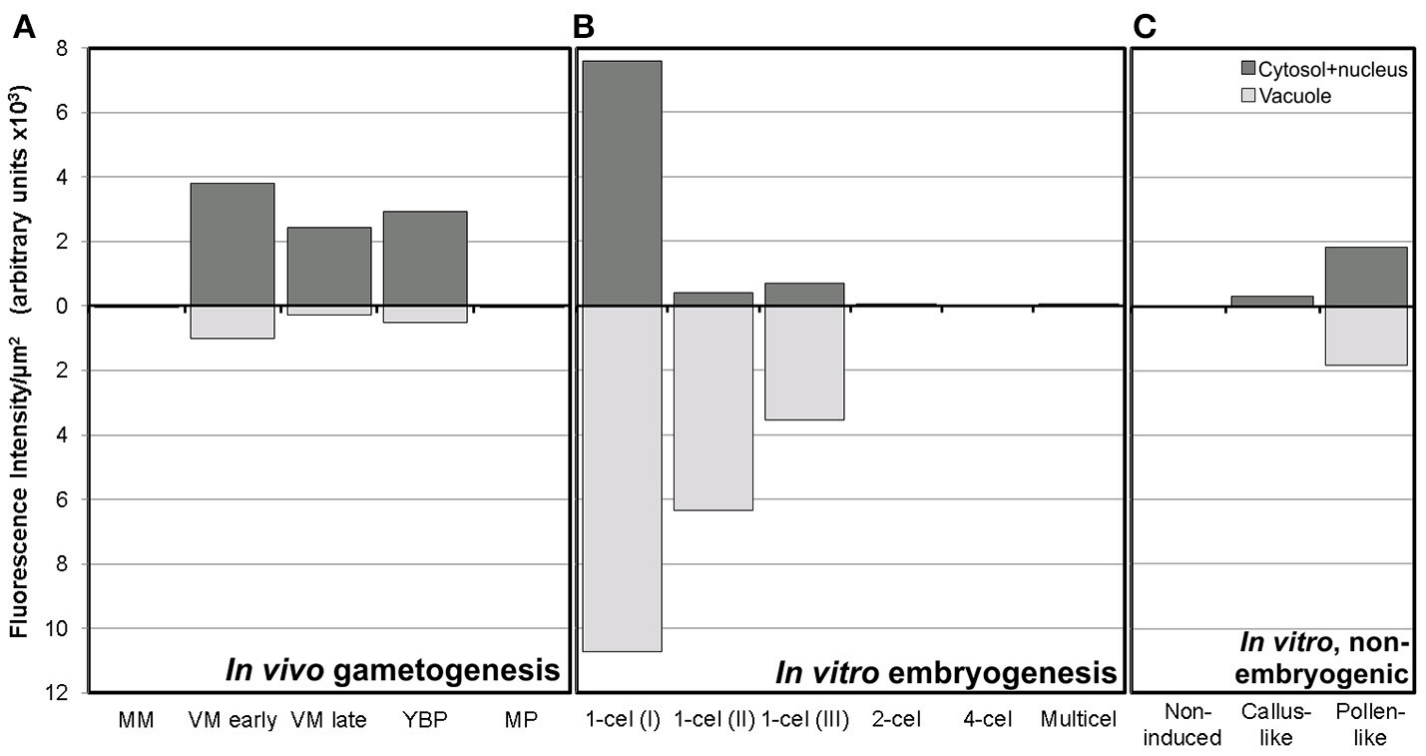
Fluorescence intensity of FF staining in cytoplasm and vacuoles of rapeseed cells at different stages during *in vivo* gametogenesis **(A)**, *in vitro* embryogenesis **(B)**, and non-embryogenic *in vitro* development **(C)**.

**Figure 5:**
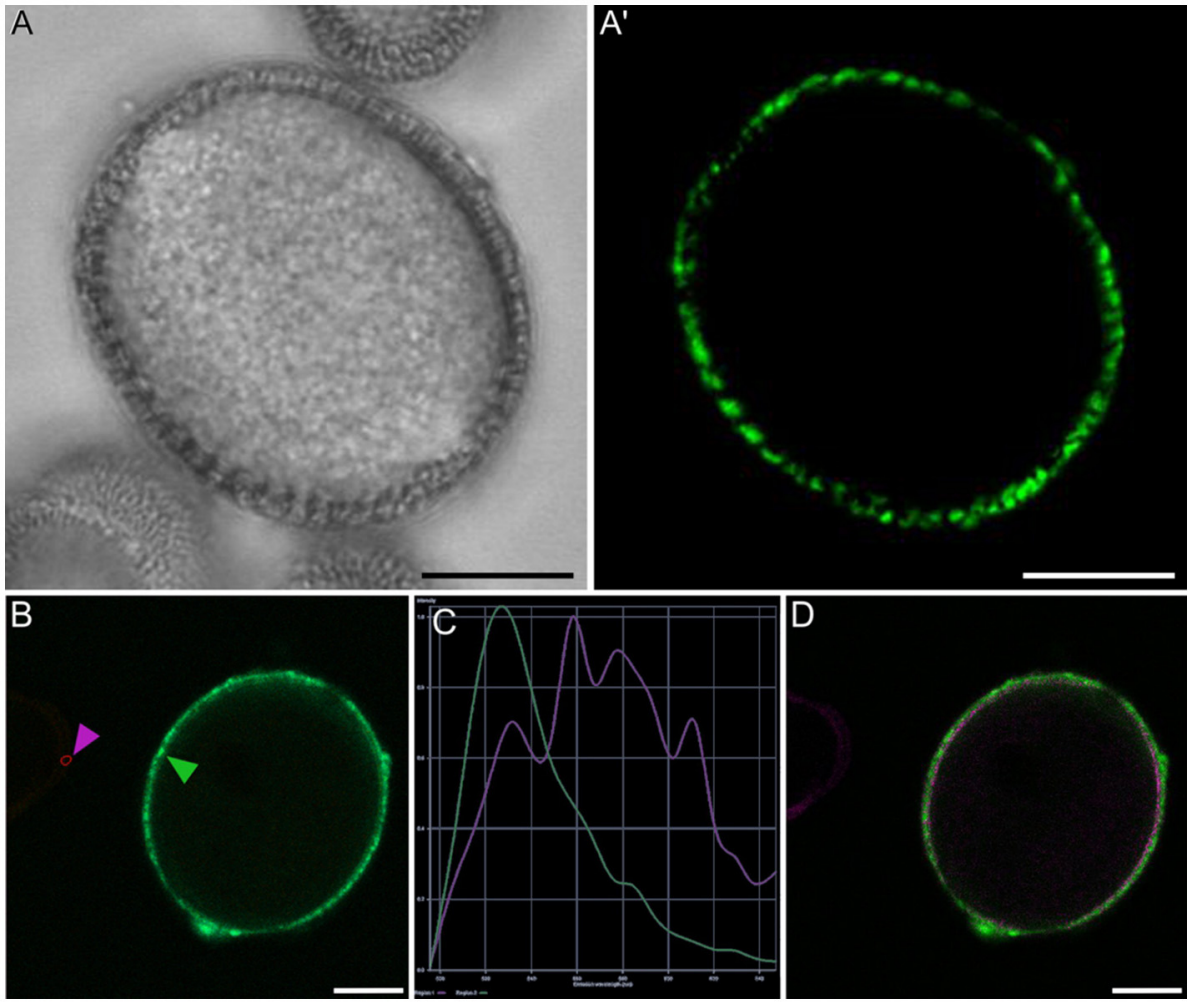
Rapeseed mature pollen grain stained with FF and observed under phase contrast **(A)** and fluorescence **(A**′**)**. Panels **(B–D)** show the analysis of fluorescence emission spectra of exine autofluorescence and FF. **(B)** Small areas were selected from mature pollen exine (green arrowhead) and microspore exine (purple arrowhead), **(C)** Fluorescence emission spectrum of pollen exine (green) and microspore exine (purple), **(D)** Areas with different emission spectra are represented in coded colors, allowing the identification of FF staining (green) and exine autofluorescence (purple). Note that in the pollen wall, FF staining and exine autofluorescence are overlaid. Bars: 10 μm.

A representative example of the calcium distribution at each stage during microsporogenesis and microgametogenesis is shown in Figure 3. Observations were remarkably consistent for each stage. Tetrads (Figures 3A,A′) and young microspores just released from the tetrad (Figures 3B,B′) presented a very low calcium signal, principally located in few, small cytosolic foci, likely corresponding to cytoplasmic organelles. Mid microspores showed no detectable calcium signal (Figures 3C,C′). At the onset of microspore vacuolation, calcium signal accumulated in the cytosol and nucleus, while no detectable signal was found in the vacuole (Figures 2A,A′). Late unicellular, vacuolate microspores (Figures 3D,D′) also showed nuclear-cytosolic signal, but brighter than in the previous stage. As in the previous stage, the large, central vacuole was devoid of fluorescence, but in this stage, the nuclear region appeared slightly brighter than the cytosol. The highest signal intensity was observed after the first pollen mitosis, in early binucleated pollen (Figures 3E,E′; Movie S1). In this stage, an intense fluorescence was observed in the cytosol and even more intense in the nuclei, but not in the mid-sized vacuoles that resulted from fragmentation of the large vacuole. In the mid pollen grain (Figures 3F,F′) calcium staining was in general less intense, but it was observed again in the cytosol and the centrally positioned nuclei. The small and numerous vacuoles and the starch granules typical from this pollen stage showed no detectable signal. In order to confirm our qualitative observations, we calculated for all the cells studied at each stage, the average FF fluorescence intensity/μm^2^ in all the cell area and in each of the identifiable cell regions. As seen in Table 1 and in Figure 4A, this analysis confirmed that at the stages of microspore vacuolation and first pollen division, calcium signal strongly increases in the nuclear-cytosolic regions, staying very low in vacuoles. In mature pollen grains (Figures 5A,A′), a faint, barely detectable signal was observed inside the pollen grain, in line with the progressive decrease observed after the first pollen mitosis. However, numerous bright foci were detected in the exine, outside the cell. To exclude the possibility of exine autofluorescence, a known and well-documented phenomenon (Roshchina, 2012), we analyzed both emission spectra. Spectral imaging as described in Section Materials and Methods was carried out in preparations containing both microspores and mature pollen grains and exposed to the same excitation conditions. Small regions were selected in the pollen and microspore exine (Figure 5B, green and purple arrowheads, respectively), and the fluorescence emission spectra of the selected areas were analyzed. As seen in Figure 5C, the pollen exine spectrum (green curve) presented the characteristic emission spectrum of FF (one neat peak with a maximum in 525 nm), whereas the microspore exine spectrum (purple curve) showed multiple peaks at different wavelengths, typical of autofluorescence. Indeed, when the linear unmixing procedure provided by the LAS AF software was used to assign different color to areas with different emission spectrum, both signals perfectly overlaid (Figure 5D). Therefore, the exine signal observed in mature pollen grains was not due to autofluorescence.

**Table 1:** Average intensities of FF fluorescent staining for different regions of microspores/pollen during *in vivo* development.

**Stage**	**Vacuoles**	**Cytosol + nucleus**	**Exine**
Mid microspore	0.01 ± 0.01	0.01 ± 0.01	n.d.
Early vacuolate microspore	1.00 ± 1.11	3.80 ± 1.75	n.d.
Late vacuolate microspore	0.28 ± 0.39	2.45 ± 1.65	n.d.
Young bicellular pollen	0.52 ± 0.29	2.94 ± 2.27	n.d.
Mature pollen	0.03 ± 0.02	0.03 ± 0.02	4.04 ± 1.63

*Fluorescence intensity is given in arbitrary units × 10^3^/μm^2^*.

Finally, we decided to check out whether the different FF fluorescence we observed in different stages was actually due to the presence of different calcium levels, or instead, was due to a different dye uptake in different stages. For this we used FDA, an acetoxymethyl ester like FF that passively enters the cell and interacts with intracellular esterases, exactly as FF does. However, unlike FF, FDA is not sensitive to calcium nor to anything else. FDA de-esterification directly releases fluorescein. Therefore, similar dye uptakes at different stages during microsporogenesis and microgametogenesis should give rise to similar fluorescence levels. Figure S3 shows that from the tetrad to the mature pollen stage, rapeseed microspores and pollen at different developmental stages presented an equivalent fluorescence pattern, characterized by a homogeneous staining of the nucleus, cytoplasm and cytoplasmic organelles excluding vacuoles. A similar FDA staining pattern implies a similar dye uptake. In other words, the different FF fluorescence observed in different stages was not due to a different, stage-specific dye uptake, but to different, stage-specific calcium levels.

In conclusion, we showed that during *in vivo* development, calcium levels increased progressively in the cytosol and nucleus (but not in vacuoles) from the tetrad stage to the late vacuolate microspore stage, first as discrete foci and then as a dispersed signal that reached a maximum at the young pollen stage. From then on, calcium levels progressively decreased to become barely detectable in the cytosol of mature pollen. However, an intense calcium signal was observed at this stage in the pollen exine.

### Calcium distribution during induction of microspore embryogenesis in rapeseed

**Figure 6:**
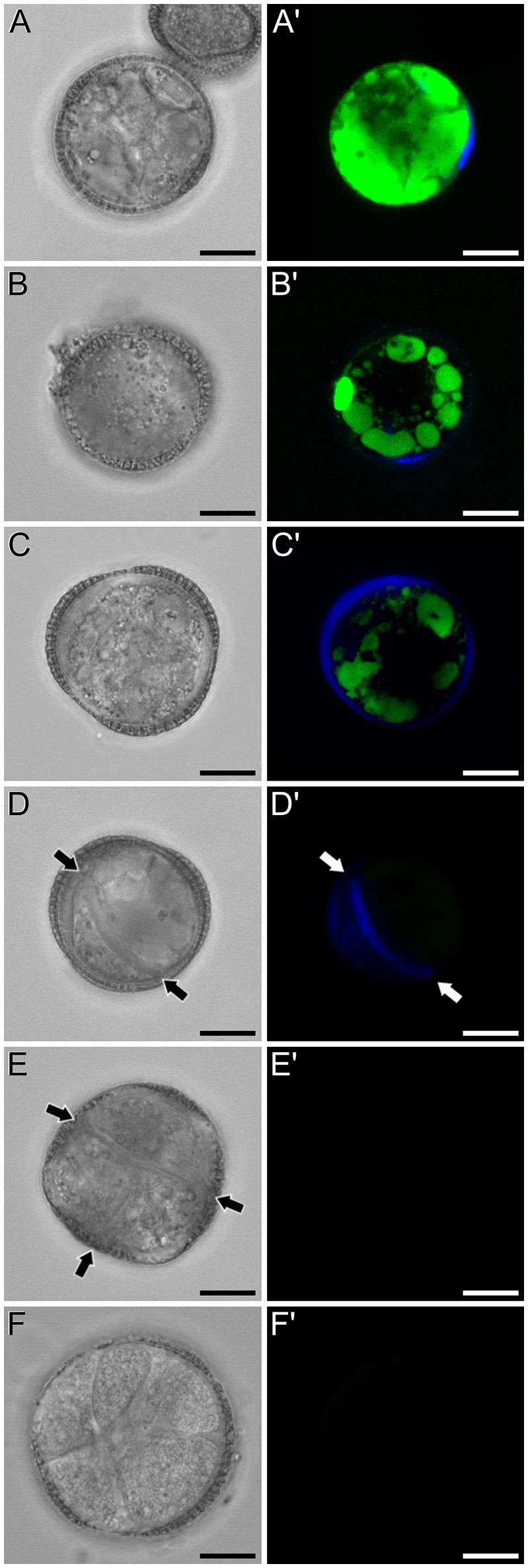
FF and aniline blue staining during *in vitro* microspore embryogenesis in rapeseed. Phase contrast **(A–F)** and fluorescence **(A′–F′)** pairs of pictures are shown. **(A,A′)** Induced microspore showing high FF green signal, and few aniline blue stained regions below the intine. **(B,B′)** Microspore showing FF staining only in vacuoles, and aniline blue signal in the subintinal layer. **(C,C′)** Embryogenic microspore with decreased vacuolar FF signal and fully surrounded by aniline blue-positive subintinal layer. **(D,D′)** Embryogenic microspore with a clear aniline blue-stained cell division (arrows) and very low level of FF staining. **(E,E′)** Four-celled embryogenic structure (arrows indicate inner cell walls), without detectable fluorescent signal. **(F,F′)** Multicellular embryogenic structure without detectable fluorescent signal. Bars: 10 μm.

Next, we studied the intracellular levels and distribution of calcium signal in rapeseed microspores isolated and *in vitro* induced to embryogenesis. To identify them we used different morphological markers (size, shape, cytoplasmic appearance, etc.), being the most evident the presence of internal cell walls. In order to identify microspores committed to embryogenesis but still not divided, we stained cells in parallel with anilin blue to identify the development of the callose-rich subintinal layer, described as an early marker of embryogenic commitment (Parra-Vega et al., 2015b). One day after induction (Figures 6A,A′), we found many cells where the intracellular calcium signal increased dramatically with respect to that found in *in vivo* isolated microspores. These cells also presented aniline blue staining at discrete peripheral regions, indicative of the onset of subintinal layer formation. The calculation of the average FF fluorescence intensity/μm^2^ showed that in these cells [1-cel (I) in Table 2 and Figure 4B], in addition to a ~2.5× increase in the nuclear-cytosolic signal, these microspores accumulated signal in vacuoles at levels considerably higher (~19×) than during *in vivo* development. Indeed, the most striking difference between *in vivo* and *in vitro* development was the massive internalization of calcium signal to vacuoles. In 1 day-old cultures we also observed microspores with larger peripheral regions stained with aniline blue, indicating a later stage in subintinal layer formation. Interestingly, these cells [1-cel (II) in Table 2 and Figure 4B] showed a decrease in the nuclear-cytosolic calcium signal, being nearly all the signal concentrated in vacuoles, now located at the cell periphery (Figures 6B,B′; Movie S2). A third type of unicellular microspores [1-cel (III) in Table 2 and Figure 4B] was fully surrounded by aniline blue-positive subintinal layer and showed a decrease in vacuolar signal (Figures 6C,C′), suggesting that as the cell progresses in embryogenesis, calcium signal decreases. This notion was confirmed in embryogenic structures with clearly visible cell divisions (Figures 6D,D′), where vacuoles showed no detectable signal, and the nuclear-cytosolic signal dropped down to very low levels. Four-celled (Figures 6E,E′) and multicellular embryogenic microspores (Figures 6F,F′) from 6-day old cultures, where the subintinal layer is already dismantled (Parra-Vega et al., 2015b), followed this trend, with almost no detectable calcium signal neither in the vacuoles nor in the rest of the cell.

**Table 2:** Average intensities of FF fluorescent staining for different regions of *in vitro*- cultured structures.

**Stage**	**Vacuole**	**Cytosol + nucleus**
1-cel (I)	10.74 ± 1.92	7.58 ± 2.55
1-cel (II)	6.36 ± 2.54	0.40 ± 0.24
1-cel (III)	3.54 ± 2.20	0.69 ± 0.87
2-cel	0.04 ± 0.08	0.04 ± 0.08
4-cel	n.d	n.d
Multicel	0.01 ± 0.01	0.01 ± 0.01
Not induced	0.01 ± 0.01	0.01 ± 0.01
Callus-like	0.05 ± 0.08	0.31 ± 0.20
Pollen-like	1.84 ± 2.12	1.84 ± 2.12

*Fluorescence intensity is given in arbitrary units × 10^3^/μm^2^*.

In light of these results, we concluded that the first signs of embryogenic commitment are accompanied by an increase in intracellular calcium levels. In particular, calcium accumulated in vacuoles, disappearing from the nucleus and cytosol. However, later embryogenic stages, when microspores start the successive division rounds, are characterized by a progressive decrease in calcium levels, reaching undetectable levels not only in the cytosol, but also in vacuoles.

### Calcium distribution in non-induced, *in vitro* cultured rapeseed cells

**Figure 7:**
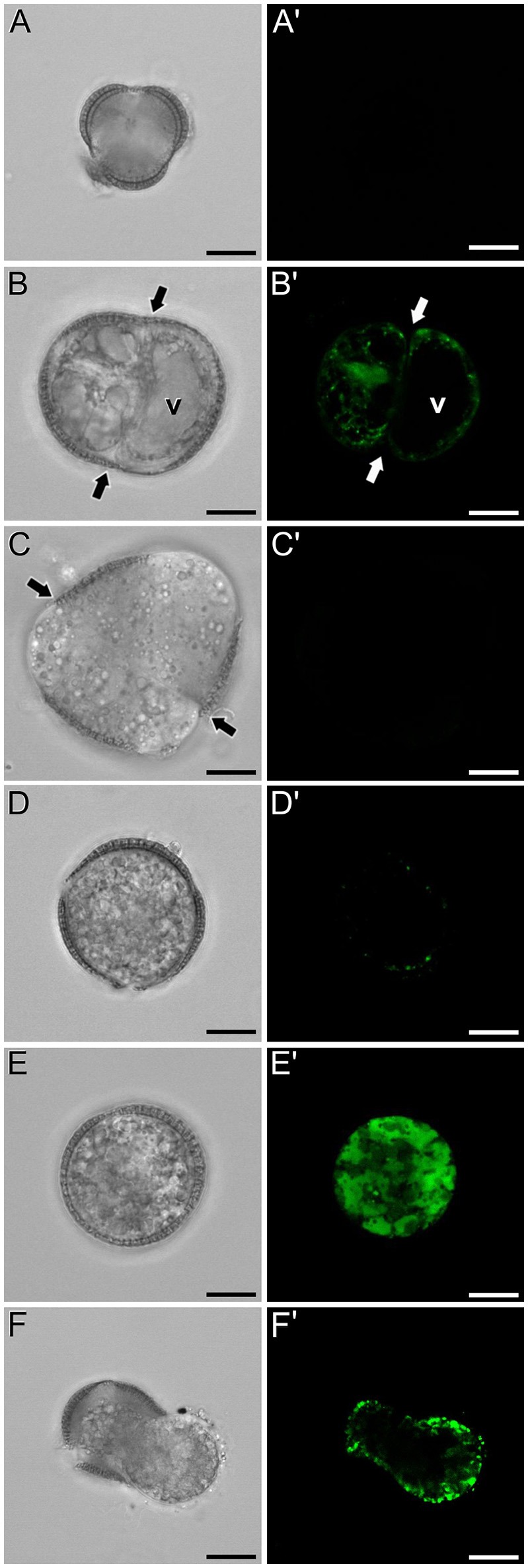
FF and aniline blue staining during non-embryogenic *in vitro* development in rapeseed. Phase contrast **(A–F)** and fluorescence **(A′–F′)** pairs of pictures are shown. **(A,A′)** Non-induced, arrested, or dead microspore. **(B,B′)** Callus-like structure with non-embryogenic cell divisions (arrows). **(C,C′)** Six day-old multicellular callus-like structure (arrows indicate inner cell walls). **(D,D′,E,E′)** Pollen-like structures with different levels of FF signal, from low and concentrated in discrete peripheral foci **(D,D′)** to intense and distributed throughout the cytosol. **(F,F′)** Pollen-like structure with broken exine resembling germinating pollen, showing FF signal in the peripheral cytosolic area. Bars: 10 μm.

In isolated microspore cultures, microspores induced to embryogenesis coexist with other forms, non-sensitive to the inductive treatment and therefore, not induced to embryogenesis. For instance, there are cells arrested or dead at different culture stages (Figure 7A), microspores induced to divide and proliferate but in a non-embryogenic manner, giving rise to disorganized, callus-like structures (Figures 7B,C), and microspores that follow a gametophytic-like pathway, becoming pollen-like grains (Figures 7D,E) with many of the features typical from pollen grains, including enlarged size, vegetative and generative nuclei, and starch granules, among others. Microspores apparently arrested or dead, not showing any sign of development, showed no detectable signal in any case (Figures 4C, Figure 7A′). Dividing cells that followed a callus-like pathway showed a pattern of FF staining remarkably different from embryogenic microspores (Figure 4C). These structures presented a faint but clearly detectable signal in the nuclear-cytosolic region (Figure 7B′), combining a dispersed pattern with the presence of some discrete foci as those observed in *in vivo* microspores. Interestingly, we detected this pattern of calcium signal in these structures even in 3 day-old cultures, a stage when calcium could not be detected in embryogenic, dividing structures. In all cases, signal was not present in vacuoles, at least at detectable levels. After 6 days of culture (Figure 7C′), however, calcium signal in callus-like structures became eventually undetectable in our conditions. Pollen-like structures presented a dual pattern of calcium distribution. Some of them presented very scarce signal, concentrated in few small, peripheral foci (Figure 7D′). Others presented abundant cytosolic signal, principally in peripheral regions (Figure 7E′). Interestingly, some of them showed a broken exine and part of the cytoplasm emerging out of the grain, resembling germinating pollen (Figures 7F,F′). In any case we could find in pollen-like structures a calcium distribution pattern similar to that found in embryogenic microspores.

### Calcium distribution during microspore and pollen development in eggplant

**Figure 8:**
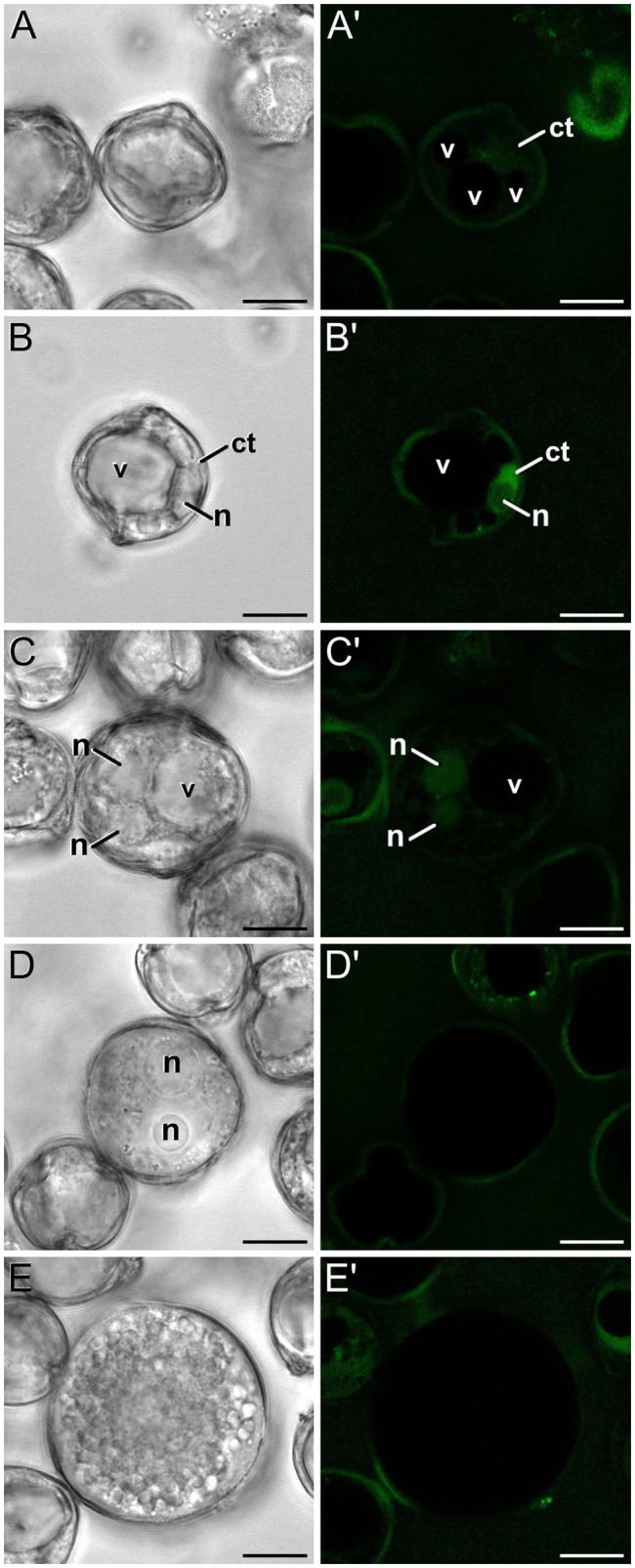
FF staining during *in vivo* microspore/pollen development in eggplant. Phase contrast **(A–E)** and fluorescence **(A′–E′)** pairs of pictures of a mid microspore **(A,A′)**, young microspore **(B,B′)**, young, just divided pollen grain **(C,C′)**, mid pollen grain **(D,D′)**, and mature pollen grain **(E,E′)** are shown. ct, cytosol; n, nucleus; v, vacuole. Bars: 10 μm.

In order to check whether the calcium profiles observed during microspore and pollen development in rapeseed are exclusive of this species or a common feature shared with other species, we studied the changes in calcium distribution in eggplant microspores and pollen. In young and mid microspores, no detectable calcium signal could be observed (data not shown). As microspores began to undergo vacuolation, calcium signal faintly accumulated in the cytosol and nucleus, while no signal at all was found in the vacuole (Figures 8A,A′). Late unicellular, vacuolate microspores (Figures 8B,B′) showed a similar but slightly brighter pattern of nucleo-cytosolic and not vacuolar signal. After the first pollen mitosis, young pollen grains (Figures 8C,C′) showed a similar profile, but being the nuclear signal brighter than in previous stages. However, in mid pollen grains (Figures 8D,D′) calcium staining was not detectable, as it was in mature pollen grains (Figures 8E,E′). As in rapeseed, bright foci were also observed in the exine, outside the cell, but at a considerably lower amounts, only one or two per exine slice (Figures 8D′,E′).

## Discussion

### Fluoforte staining is a convenient way to detect intracellular Ca^2+^ in living *in vivo* and *in vitro* cultured microspores

Despite the interest of the long pursued study of the changes in intracellular calcium levels and distribution in plant cells, this study has not been always easy. Among the different technical alternatives, the most used have been Ca^2+^-sensitive fluorescent dyes. Different dyes are commercially available, but they generally do not diffuse well into cells because at physiological pH, they are negatively charged (Grynkiewicz et al., 1985). The alternative, uncharged acetoxymethyl (AM) esters of the dyes, are able to passively enter the cytosol, where they interact with intracellular esterases that switch them to the negatively charged, Ca^2+^- sensitive form (Tsien, 1981). In plants, this approach may be limited by the presence of esterases (pectin methyl-esterases, PMEs) in the cell wall (Micheli, 2001), which hydrolyze AM-esters in the extracellular space prior to access the cytosol. However, Solis et al. (2016) found that the expression of PMEs in rapeseed vacuolate microspores, pollen grains and embryogenic microspores was very low. Thus, it seems that the presence of cell wall PMEs should not be a problem in our case. Our results confirmed it, since we were able to detect an intense signal within rapeseed microspores and pollen grains. Although at different intensities, we observed a similar calcium pattern during eggplant microspore and pollen development, which extends the range of applicability of this method beyond rapeseed. As to the presence of FF signal in the exine of mature pollen, it could be due to the presence of other, mature pollen-specific PMEs, or to the contribution of the senescing inner anther wall layers which, together with the release of calcium (Ge et al., 2007b; Qiu et al., 2009; Kuang and Liao, 2015) and of partially and non-methyl-esterified pectins to the locular fluid (Corral-Martínez et al., 2016), could be delivering pectin-associated PMEs.

We also demonstrated that the different FF fluorescence observed is not due to stage-specific differences in cell permeability or in dye uptake, because FDA, an AM-ester dye as FF, but insensitive to calcium, yielded similar fluorescence levels in all the stages observed. Another problem previously documented with the use of fluorescent calcium dyes in plant cells is intracellular compartmentalization after loading (Fricker et al., 2001). However, this strongly depends on the cell type and the dye used. For example, Bush and Jones (1987) used two AM-ester dyes, Indo-1 and Fura-2, to image Ca^2+^ in barley aleurone protoplasts. They found that while Indo-1 was not well-hydrolyzed, Fura-2 compartmentalized in the vacuole. Similarly, in rhizoid cells of *Fucus serratus*, Fura-2 was found sequestered into vacuoles and vesicles (Brownlee and Pulsford, 1988). Using FF in rapeseed microspores, we also found some degree of dye compartmentalization in the vacuole, but 1 h was needed to observe it. When observed within 15 min after mounting, intracellular calcium signal was exclusively cytosolic and nuclear. Other studies, using absolutely different approaches and species (Kong and Jia, 2004; Ge et al., 2007b; Qiu et al., 2009; Kuang and Liao, 2015; Wei et al., 2015), consistently showed a clearly cytoplasmic signal, which counts in favor of this notion too. Thus, we can assume that in *in vivo* microspores, the signal we observed corresponded to cytosolic and nuclear Ca^2+^. Similarly, we can also assume that when we see, under identical preparative conditions, cells with mostly vacuolar signal (as in embryogenic microspores), we are detecting Ca^2+^ initially located in vacuoles, and not compartmentalized. It must be noted, though, that compartmentalization prevented us from using live imaging of Ca^2+^ changes in the same microspores during the first embryogenic stages. This was the reason to take different samples for different time points, instead of observing the same cells over time. Since we kept our *in vivo* samples at 4°C to avoid the putative effect of heat on calcium levels, it could also be argued that cold might also have an effect. However, calcium oscillations due to cold shock are brief (seconds, according to Knight, 1999; White and Broadley, 2003), which implies that after a 15 min incubation, we should not observe them. In addition, the exposure of samples to different treatments and several buffers to be able to reveal FF fluorescence might preclude from obtaining a more precise picture of absolute levels under physiological conditions, which would need further investigations in near-to-physiological conditions. All this considered, the method we hereby described may not be useful to detect short calcium pulses or absolute levels under physiological conditions, but we can rely on the use of FF to detect differences in calcium levels and distribution among different stages during *in vivo* and *in vitro* microspore and pollen development.

### Species responsive to microspore embryogenesis show a particular calcium dynamics during *in vivo* development

From the tetrad stage on, cytosolic Ca^2+^ progressively increased from a very low level in tetrads up to a maximum in late microspores and young pollen grains. From that stage on, Ca^2+^ progressively disappeared, reaching in mature pollen cytosolic levels as low as those of tetrads. This pattern, characterized by a sharp peak at the microspore-pollen transition, is not frequent in other species. For example, using the potassium pyroantimonate cytochemistry for electron microscopy, in *Larix principis-rupprechtii*, calcium was almost undetectable during microspore development, whereas in pollen grains, it was only clearly observed in the pollen coat (Kong and Jia, 2004). In lettuce and oil tea, a progressive increase during microsporogenesis, a decrease during vacuolation, a second increase in young pollen grains, and a final decrease in mature pollen was described (Qiu et al., 2009). In *Uncaria hirsuta*, calcium precipitates increased as microsporogenesis proceeded, with a peak in bicellular pollen and a decrease in mature pollen (Kuang and Liao, 2015). In tobacco, calcium was detected in the cytoplasm and nucleus of vacuolate microspores and bicellular pollen, decreasing at late pollen stages (Ge et al., 2007b). Using exactly the same procedure than in rapeseed, we showed in eggplant that Ca^2+^ also concentrated in the nucleus and cytosol of vacuolate microspores and young pollen grains, but at low levels, far below those of rapeseed. As seen, a calcium peak in young pollen grains seems to be a common trend for all the species above mentioned. However, in some of them calcium levels are low in vacuolate microspores and, interestingly, there are no evidences to our knowledge of successful induction of microspore embryogenesis. In contrast, other species show remarkably high calcium levels in vacuolated microspores, and successful microspore embryogenesis has been reported. This is the case of eggplant, tobacco and rapeseed. Indeed, tobacco and rapeseed are considered as model species in terms of response to induction of microspore embryogenesis. Eggplant is considered moderately recalcitrant but inducible, and in this species, the calcium levels observed in vacuolate microspores were markedly lower than in rapeseed. These observations make us propose that the particularly high Ca^2+^ levels just at the stages more suitable for embryogenesis induction are related to their ability to undergo embryogenesis. In addition, the levels of calcium present at these stages in different species would be related to their different sensitivity to embryogenesis induction.

### The unique calcium pattern of embryogenic microspores would reflect the simultaneous occurrence of multiple stresses

In rapeseed microspores induced to embryogenesis, Ca^2+^ was found in the cytosol and nucleus at levels remarkably higher than in *in vivo* vacuolate microspores and young pollen grains. This is not surprising, since cultured microspores are suspended in a calcium-rich medium [500 mg/l Ca(NO_3_)_2_], and it is known that one of the consequences of heat shock exposure is fluidization of the plasma membrane, which makes it more permeable to cations such as Ca^2+^, among others. Thus, it seems reasonable to deduce that the dramatic increase in embryogenic microspores is due to the entry of Ca^2+^ from the culture medium. According to White and Broadley (2003), the magnitude and duration of a stress-associated calcium increase depends on the severity of the stress and the number of different stresses acting together. Rapeseed isolated microspore culture is a system where different stress sources are simultaneously applied to the same population. These stresses include, at least, a mechanical stress from isolation procedures, an osmotic stress from culture in a medium with high sucrose levels (130 g/l), and a 24 h-long heat stress at 32.5°C. In turn, they induce the production of reactive oxygen species which generate additional oxidative stress. It is known that some stresses, such as mechanical stress or cold shock induce immediate, transient Ca^2+^ short pulses, whereas heat shock, hyper-osmotic stress, and exposure to oxidative stress first elicit an immediate, short Ca^2+^ pulse and also a second, prolonged elevation that may last even hours (reviewed in White and Broadley, 2003). Our experimental conditions precluded us from detecting the first short pulse, but the extremely elevated Ca^2+^ levels observed after 24 h of *in vitro* culture would reflect the second, prolonged elevation of such biphasic calcium signature caused not only by exposure to heat stress, but also to hyper-osmotic and oxidative stress. Thus, just induced embryogenic microspores combine, simultaneously, high initial Ca^2+^ levels and a series of stress-inducing factors characterized by prolonged Ca^2+^ elevations. Such unique combination would be the cause of their disparate Ca^2+^ levels.

### The unique calcium pattern of embryogenic microspores might be involved in the developmental switch

In this cellular scenario, it is tempting to speculate with the consequences of this unique Ca^2+^ perturbation. First, it might be related to autophagy. We demonstrated that induction of embryogenesis in rapeseed is tightly associated with massive autophagy and excretion processes involving the formation of autophagosomes and plastolysomes (Corral-Martínez et al., 2013; Parra-Vega et al., 2015a). On the other hand, a clear link between calcium signaling and autophagy induction through regulation of PPP3/calcineurin (a calcium-dependent phosphatase) was recently demonstrated in mammal cells (Medina et al., 2015). Although possible, this hypothesis is still weak, since a similar link between calcium and autophagy is still to be demonstrated in plants. However, the links between Ca^2+^ perturbations and plant embryogenesis induction are significantly stronger and well-founded. It is widely accepted that during zygotic embryogenesis, calcium is needed for egg cell activation. In both plants and animals, the initial step of egg activation involves dramatic Ca^2+^ increase and oscillations. In mouse oocytes, it was demonstrated that the promotion of calcium uptake with the A23187 ionophore was sufficient to activate nearly 50% of the treated oocytes (Nakasaka et al., 2000). In plants, there are multiple evidences of Ca^2+^ increases upon fertilization that point to a key role of calcium in egg cell activation (reviewed in Ge et al., 2007a). The most notable example comes from maize, where the first events of the embryogenic program can be induced just by triggering Ca^2+^ influx (Antoine et al., 2001). In other embryo-forming processes, the involvement of calcium is similar. Experimentally elevated Ca^2+^ levels were found to stimulate somatic embryogenesis in *Coffea canephora* (Ramakrishna et al., 2012) and carrot (Takeda et al., 2003), where initiation of somatic embryogenesis was found to coincide with a rise in the level of cytosolic Ca^2+^ (Timmers et al., 1996). Although, there is no evidence for an androgenesis-specific calcium signature, the calcium pattern we hereby describe is the most detailed description of calcium dynamics during the first stages of MDE induction, and presents remarkable similarities with calcium dynamics in other embryo-forming processes. Since it is widely accepted that explicit calcium perturbations produce specific signatures which trigger defined physiological responses (reviewed in White and Broadley, 2003), we speculate that the unique combination of elevated initial Ca^2+^ levels and additional stresses in embryogenic microspores would be somehow mimicking the specific calcium perturbations that appear to initiate embryogenesis in egg and somatic cells under defined circumstances.

Interestingly, calcium increase is not the only common link with other embryogenic processes. The formation of a callose-rich layer surrounding the cell as soon as it acquires embryogenic identity is a common feature of embryogenic microspores (Parra-Vega et al., 2015b), somatic embryos (Maheswaran and Williams, 1985; Dubois et al., 1991; You et al., 2006) and zygotic embryos as well (Jensen, 1968; Williams et al., 1984). The similarities between calcium patterns would add to the growing body of evidences that relate the different embryogenic pathways not only ant the genetic level, but also at the cellular and physiological levels.

### Vacuolar calcium internalization could help cells prevent toxicity of calcium excess

An additional difference with *in vivo* microspores was the massive internalization of calcium in vacuoles during the inductive treatment. It is known that there is a maximal concentration and duration beyond which, prolonged increases in cytosolic Ca^2+^ become toxic and even lethal for cells (White and Broadley, 2003). Indeed, sustained high Ca^2+^ levels were shown involved in programmed cell death during both normal development and abnormal situations such as hypersensitive responses to pathogens (Levine et al., 1996). Thus, vacuolar storage of calcium excess in embryogenic microspores may be a mechanism to keep calcium homeostasis under control and therefore, avoid calcium toxicity or death induction. It is interesting to note that the presence of markers of embryogenic commitment such as the callose-rich subintinal layer (Parra-Vega et al., 2015b) was inversely related to the presence of Ca^2+^ in vacuoles. A callose-rich, impermeable wall may constitute an effective barrier against Ca^2+^ influx across a plasma membrane permeabilized during heat shock. Therefore, as the subintinal layer grows and covers progressively more plasma membrane, it will be less necessary to store Ca^2+^ excess in vacuoles. In line with this, once cells were transferred to 25°C, no Ca^2+^ was detected in vacuoles, reaching a situation similar to that of young microspores or mature pollen. In conclusion, the storage of calcium in vacuoles during heat shock exposure seems to be a cellular response to reduce excessive concentration to safe cytosolic levels.

### Stress-induced calcium perturbations are not the only players involved

A remarkable feature of microspore cultures is that all microspores are initially exposed to the same *in vitro* conditions, but not all adopt the same developmental pathway nor present the same calcium patterns, as we demonstrated hereby. Embryogenic microspores showed a dramatic rise during the first stages, accumulating most of the Ca^2+^ in vacuoles, whereas callus-like structures presented almost no calcium increase, being always cytosolic, not vacuolar. In turn, pollen-like structures showed either very scarce signal, which might indicate pollen latency, or abundant peripheral signal associated in some cases to morphological evidences of pollen germination. *In vivo*, this calcium distribution pattern has been associated to germinating pollen (Ge et al., 2007a), which confirms the pollen-like behavior of these *in vitro*-induced structures. In summary, we showed three defined calcium patterns associated to three developmental fates, all triggered in adjacent cells exposed to identical stress conditions. Thus, the question arises as to why there are different responses to identical stimuli? First, it is possible that each response (embryo-like, callus-like, or pollen-like) comes from microspore/pollen grains at slightly different developmental stages, and therefore with different calcium levels. Second, and assuming that all microspores are at the same developmental stage, this phenotypic plasticity may be explained because calcium levels are not the only players involved in the responses. According to Gilroy and Trewavas (2001), the levels and activity of Ca^2+^ sensors and target proteins, among other elements, are also important, and they may not be the same in all cells, probably due to subtle physiological differences (even being at the same microspore stage) which lead to minute differences in transcript, protein, and/or enzyme profiles. This is why identical stimuli may give rise to different Ca^2+^ perturbations which, in turn, may lead to different developmental fates in two adjacent microspores.

## Author contributions

AR generated the experimental work, analyzed the results, and contributed to manuscript confection. AC contributed to the generation of experimental work. JS designed the experiments, supervised them, analyzed the results, and confectioned the manuscript.

### Conflict of interest statement

The authors declare that the research was conducted in the absence of any commercial or financial relationships that could be construed as a potential conflict of interest.

## Supplementary material

Movie S1 Early binucleated rapeseed pollen, just after the first pollen mitosis, stained with FF.

Movie S2 Embryogenic rapeseed microspore from a 1 day-old microspore culture, stained with FF and aniline blue.

Figure S1 Merged phase contrast and fluorescence images of a rapeseed lateral root tip stained with 0.1 g/l FF. Bar: 40 μm.

Figure S2 Freshly isolated (non-induced) rapeseed microspore and pollen mixtures, stained with different FF working concentrations. Phase contrast **(A–D)** and fluorescence **(A′–D′)** pairs of pictures are shown. **(A,A′)** Control with no FF staining. **(B,B′)** Staining with 0.05 g/l FF. **(C,C′)** Staining with 0.1 g/l FF. **(D,D′)** Staining with 0.2 g/l FF. Bars: 40 μm.

Figure S3 FDA staining during *in vivo* microspore/pollen development in rapeseed. Phase contrast **(A–G)** and fluorescence **(A′–G′)** pairs of pictures of a tetrad **(A,A′)**, young microspore **(B,B′)**, mid microspore **(C,C′)**, vacuolate microspore **(D,D′)**, young bicellular pollen **(E,E′)**, mid pollen grain **(F,F′)**, and mature pollen grain **(G,G′)** are shown. Bars: 10 μm.
