# Supplementary figures and images for: Dynamics of Calcium during In vitro Microspore Embryogenesis and In vivo Microspore Development in Brassica napus and Solanum melongena
Source: Front Plant Sci. 2017 Jul 7;8:1177. doi: 10.3389/fpls.2017.01177 (PMC5500647; doi:10.3389/fpls.2017.01177)

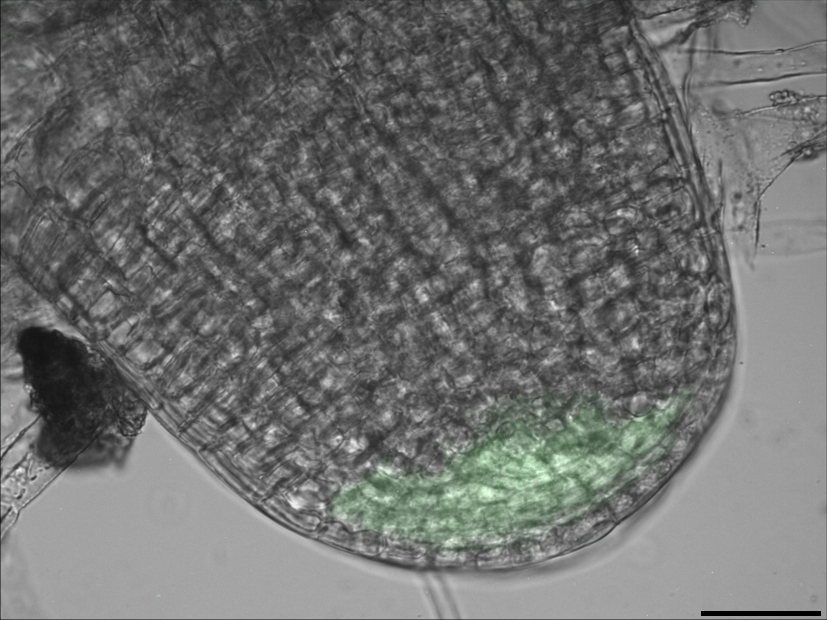

Supplement: Figure S1 — Merged phase contrast and fluorescence images of a rapeseed lateral root tip stained with 0.1 g/l FF. Bar: 40 μm. [file Image1.JPEG]

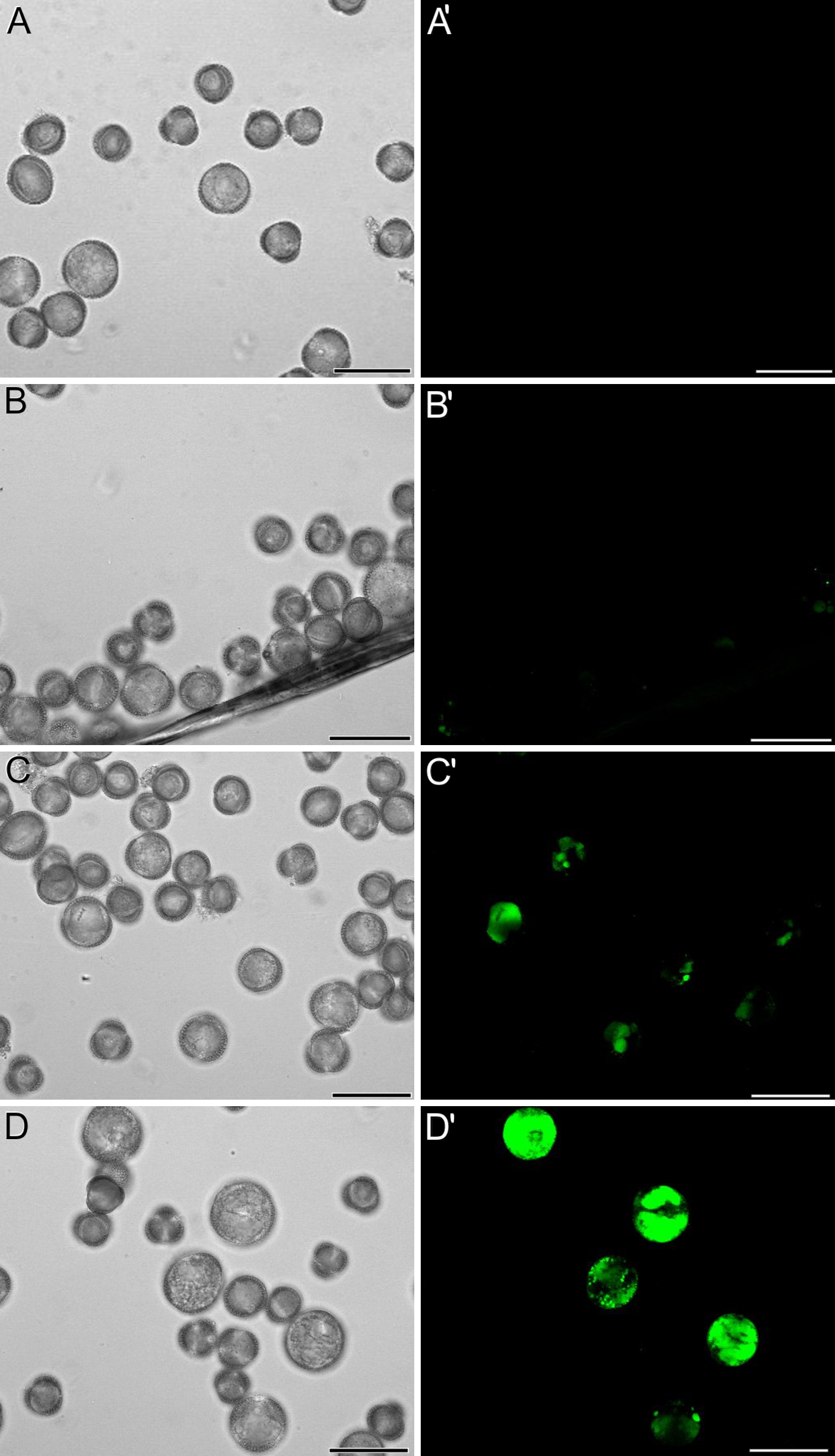

Supplement: Figure S2 — Freshly isolated (non-induced) rapeseed microspore and pollen mixtures, stained with different FF working concentrations. Phase contrast (A–D) and fluorescence (A′–D′) pairs of pictures are shown. (A,A′) Control with no FF staining. (B,B′) Staining with 0.05 g/l FF. (C,C′) Staining with 0.1 g/l FF. (D,D′) Staining with 0.2 g/l FF. Bars: 40 μm. [file Image2.JPEG]

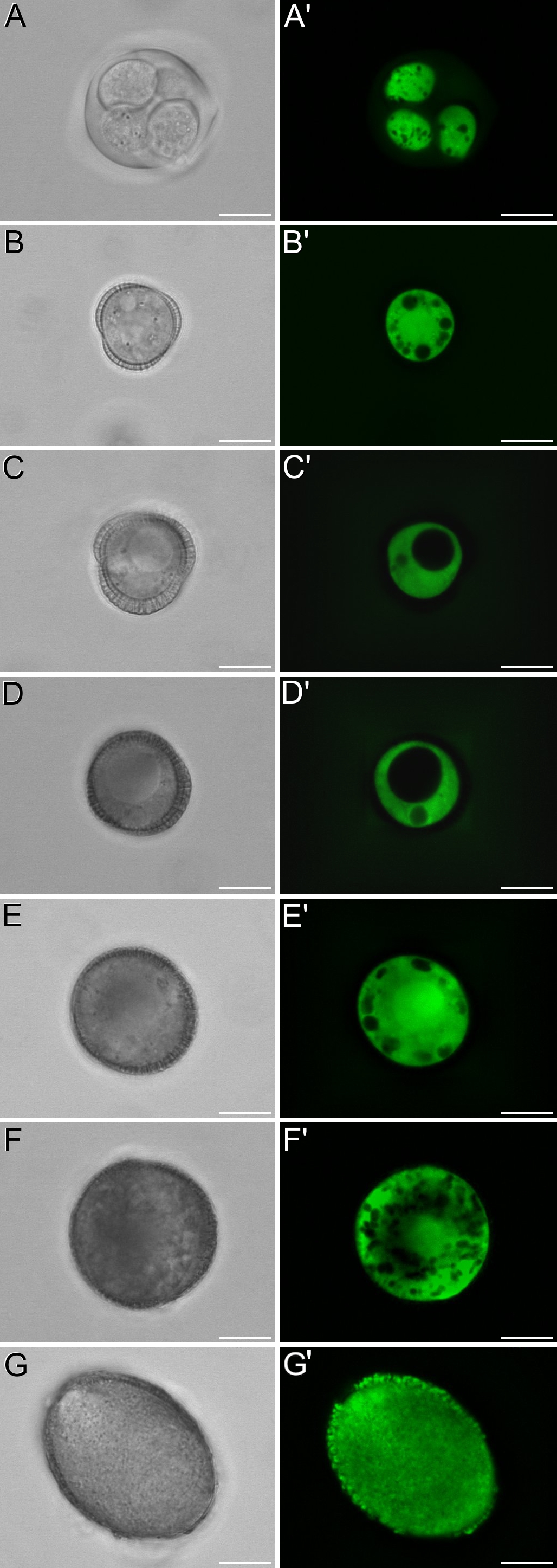

Supplement: Figure S3 — FDA staining during in vivo microspore/pollen development in rapeseed. Phase contrast (A–G) and fluorescence (A′–G′) pairs of pictures of a tetrad (A,A′), young microspore (B,B′), mid microspore (C,C′), vacuolate microspore (D,D′), young bicellular pollen (E,E′), mid pollen grain (F,F′), and mature pollen grain (G,G′) are shown. Bars: 10 μm. [file Image3.JPEG]
